# A preference‐integrated optimization system for medical physics shift scheduling

**DOI:** 10.1002/acm2.70743

**Published:** 2026-08-14

**Authors:** Benjamin S. Rosen, Zheng Zhang, Karolyn M. Hopfensperger, Kelly C. Paradis, Choonik Lee, Lana Critchfield, Ikechi Ozoemelam, Charles K. Matrosic, Christopher Kehayias, Scott W. Hadley, James M. Balter, Claire Zhang, Lise Wei, Seyyedeh Azar Oliaei Motlagh, Alexandar Moncion, Dale W. Litzenberg, Cory Knill, Maria G. Ditman, Elizabeth L. Covington, Charles S. Mayo, Sean P. Devan, Martha M. Matuszak, Joann I. Prisciandaro

**Affiliations:** ^1^ Department of Radiation Oncology University of Michigan Ann Arbor Michigan USA

**Keywords:** clinical operations, efficiency, workflows

## Abstract

**Background:**

Clinical shift scheduling for medical physicists is challenging given multidisciplinary roles and competing clinical, research, and service demands. Manual workflows often lack the flexibility and transparency needed for this complex field.

**Purpose:**

To develop, deploy, and evaluate a preference‐driven, optimization‐based scheduling platform for an academic radiation oncology medical physics division.

**Methods:**

Using a graphical user interface, each physicist encoded half‐day shift slots as unavailable (0), available but not preferred (0.5), available (1), or preferred (2) for each two‐month scheduling block over two years of clinical use. Shift requirements were defined across ten service categories with weighted effort units, and individual targets were adjusted for non‐clinical effort (e.g., research, teaching, administrative service) and planned time off. Scheduling was formulated as a constrained binary integer optimization problem, with coverage, sequencing, and availability constraints, solved using a genetic algorithm to minimize a preference‐based fitness function. Final schedules were exported to clinical calendars. Retrospective evaluation computed descriptive statistics per scheduling period and overall, including the distribution of assigned shifts by preference category and concordance between assignments and stated availability/preferences. Qualitative feedback from users and scheduling leads was collected to further assess operational impact.

**Results:**

From January 2024 through February 2026, 4980 shifts were assigned across fifteen scheduling blocks for 23 physicists. Across 25,796 preference entries, 95% of assignments matched stated preference and availability, with non‐preferred assignments accounting for only 5% of total assignments. All clinical coverage requirements were met, and no staff were scheduled for shifts marked as unavailable. Manual interventions were rare, and user feedback indicated improved transparency, satisfaction, and reduced administrative burden.

**Conclusions:**

Preference‐integrated optimization reliably produced feasible, clinically robust schedules for medical physics shift scheduling. This approach is readily implementable and supports efficient, equitable scheduling in multidisciplinary settings.

## 1 INTRODUCTION

Medical physicists play a vital role in healthcare, ensuring the safe and effective delivery of radiation therapy, among many other responsibilities. Their expertise spans a wide range of subspecialties, including quality assurance for therapeutic and imaging equipment, treatment planning, dosimetry, and specialized procedures like brachytherapy.^1^ This multifaceted role requires medical physicists to operate across different locations, manage complex technical systems, and collaborate closely with other healthcare professionals to optimize patient care. Beyond their clinical duties, physicists may also need to balance a diverse set of professional responsibilities across four broad areas: clinic, research, education, and service.^2^ The intense workload, varied responsibilities, and irregular hours contribute to high levels of stress and burnout among physicists, impacting their well‐being and job satisfaction.^3^ Relatedly, recent studies have identified scheduling workflows as a key determinant of both job satisfaction and professional sustainability.^4^ Therefore, flexible and responsive scheduling solutions are an important component of the practice of medical physics to maintain a motivated and productive workforce capable of meeting the complex demands of this field.

The study of workforce scheduling, which encompasses complex multi‐objective personnel scheduling problems, has been examined extensively across various sectors. The “nurse scheduling problem”^5^, ^6^ is a well‐known example, illustrating the intricacies of aligning staff availability with organizational demands. Applications range widely from healthcare settings to airline crew rostering and military personnel readiness, with each domain presenting unique challenges and algorithmic diversity.^7^, ^8^ Recent healthcare scheduling literature has expanded to include anesthesia, emergency medicine, surgery, rehabilitation therapy, and broader hospital personnel planning.^9^, ^10^, ^11^, ^12^, ^13^, ^14^, ^15^, ^16^, ^17^ These studies have addressed challenges such as variable clinical workload and robust scheduling,^9^, ^12^ time‐varying productivity and decision support,^10^, ^14^, ^15^ staff satisfaction,^13^ and fairness or equity in rostering.^9^, ^11^, ^16^, ^17^

However, medical physics scheduling introduces additional implementation‐specific constraints, including service‐specific expertise, full‐time equivalent (FTE)‐weighted clinical assignments clinical assignments, integration of academic and non‐clinical effort, and frequent changes in site‐ or modality‐specific coverage. Genetic algorithms (GAs) are population‐based, metaheuristic optimization methods that iteratively evolve candidate solutions through selection, crossover, and mutation.^18^ Beddoe and Petrovic^19^ used a GA to select and weight features for personnel rostering in a highly constrained nurse scheduling environment. The present work addresses the medical physics scheduling gap by translating these requirements into a clinically deployed, preference‐integrated optimization workflow for medical physics shift scheduling. The proposed model allows each physicist to express their preferred working days and coverage types, thereby aligning shift assignments more closely with individual preferences and subspecialty expertise. By accommodating these preferences, our approach aims to enhance work‐life integration and improve overall departmental efficiency across multiple sites with diverse operational demands. Moreover, by automating the complex scheduling of varied hours required in medical physics, our optimization model provides an added benefit by efficiently handling unpredictable scheduling needs. This flexibility ensures that crucial tasks are performed in a timely way without overburdening individual physicists, thus supporting a sustainable work environment. This paper presents and evaluates the effectiveness of our preference‐integrated scheduling model. We explore how this approach can be leveraged to optimize shift schedules in medical physics departments, offering valuable insights for similar applications in other multidisciplinary healthcare settings.

## 2 MATERIALS AND METHODS

### 2.1 Overview of scheduling needs

The clinical arm of the University of Michigan Department of Radiation Oncology Medical Physics Division consists of seventeen academic medical physicists, two medical physics staff members, and four clinical medical physics residents, each playing an integral role in patient care. We support a mid‐sized, tertiary radiation oncology department that is highly engaged in clinical service, research, and education. Collectively, our team facilitates approximately 850 patient treatments weekly across two locations. These sites are equipped with six standard linear accelerators, two computed tomography simulators, a magnetic resonance imaging simulator, a high‐dose‐rate (HDR) brachytherapy afterloader, and an online adaptive radiotherapy treatment system. Our clinical services may be divided into 10 categories, including general dosimetry support, special medical physics consultations (SMPCs), comprehensive machine coverage at our main and community practice site, stereotactic radiosurgery (SRS) and stereotactic body radiotherapy (SBRT) coverage, and brachytherapy coverage, covering both HDR treatments as well as low‐dose‐rate (LDR) eye plaques and Y‐90 radioembolization cases. The number of brachytherapy modalities necessitates both primary and secondary coverage slots within the daily schedule. Table 1 presents a comprehensive list of clinical service categories and their corresponding shift weights, which are standardized to 1 FTE day, generally approximating an 8‐h workday. Some physicists specialize in areas such as brachytherapy or dosimetry, while others have broader expertise, supporting a wider range of clinical services. In addition to clinical work, medical physics faculty engage in research, education, and professional service at the institutional, regional, national, and international level, with numerous related commitments often scheduled during normal working hours. The challenge of scheduling all these services while accommodating time for other commitments is a common aspect in this field.^4^

**TABLE 1:** Clinical service and relative FTE shift weight.

Clinical service	Shift weight
General Dosimetry	1.0
General Dosimetry + Re‐irradiation Consults	1.25
Machine Coverage—Main Campus (half day)	0.8
Machine Coverage—Community Practice (full day)	1.0
SBRT/SRS (half day)	0.75
Brachytherapy, Primary	1.0
Brachytherapy, Secondary	0.5
Intravascular Brachytherapy	0.4
Afterhours QA	1.0
Afterhours On‐call (full week)	0.1

### 2.2 Prior scheduling method

Previously, shift assignments in our physics division began with the lead physicists for brachytherapy, dosimetry, general machine coverage, and special procedures (e.g., SRS and SBRT) services independently determining the number of shifts required for each area, primarily based on past coverage patterns. These shift allocations were then sent to the clinical director, who assigned shifts counts to individual faculty members according FTE assignments for each clinical service. The assignment of FTEs followed a framework like that described by Kim et al.,^20^ which aims to align each physicist's shift load with their designated fractional clinical appointment. Further adjustments were incorporated when accommodating the onboarding of new faculty, staff, and residents. Once the total shift counts for all services were finalized, faculty and staff were invited to select shifts on a first‐come, first‐served basis using a shared Outlook calendar, followed by the medical physics residents several days to a week later. The process required frequent emails, manual swapping of shifts, and informal negotiations among participants to finalize the coverage schedule.

### 2.3 Challenges and rationale for change

The previous scheduling model presented several notable limitations. First, clinical schedules were developed independently by four separate service leads, which meant that the overall distribution of workload among faculty and staff was not systematically reviewed and non‐clinical effort was difficult to accurately incorporate into individual shift counts. This approach led to inequities in clinical coverage, with some individuals, particularly those supporting multiple service areas, assigned a disproportionately high number of shifts. Second, the first‐come, first‐served manual sign‐up process gave an inherent advantage to those who happened to access email notifications first, allowing them to select preferred shifts. Paradoxically, those who were actively engaged in clinical activities were left with fewer and often less desirable choices, frequently requiring shift swaps or acceptance of suboptimal assignments. Physicists who were out of the office during the sign‐up period were compelled either to log in remotely or accept undesirable shift assignments. Another significant limitation was the inadequate handling of planned time off. When team members were scheduled to be out for extended periods, service leads often were not aware of these absences during the initial scheduling phase. As a result, all of that individual's required shifts had to be compressed into the remaining available days, frequently leading to infeasible or overly burdensome schedules.

These issues collectively contributed to disparities in workload. The resulting administrative burden also fell largely on the clinical director and service leads, who were often required to resolve conflicts and rearrange coverage nearly continuously, due to the compounding nature of filling scheduling gaps. These persistent challenges motivated the search for a more equitable, transparent, and preference‐driven scheduling solution.

### 2.4 Standardized FTE assignments

To address the challenges of inequitable and fragmented scheduling, the division implemented a centralized system managed collaboratively by the Clinical Director, Division Director, and selected faculty volunteers. All scheduling is now coordinated through a comprehensive spreadsheet that unified the assignment process across the entire team. This spreadsheet consolidates each team member's clinical FTE alongside other professional responsibilities, such as funded research, special projects, educational roles, and administrative duties. Each category of non‐clinical effort is itemized as a percentage effort or time allocation and accounted for, and a specific clinical FTE target is calculated for every individual. Clinical shift assignments are then reduced commensurately to reflect these non‐clinical responsibilities. The targets are reviewed and updated every two months to reflect current commitments and planned time off, ensuring workload distribution accurately represents each person's evolving professional and personal obligations. To facilitate objective scheduling, each clinical service is assigned a weighted value representing its typical effort and time commitment (see Table 1). For example, general dosimetry shifts carry a weight of 1.0, while specialized procedures such as SBRT/SRS are weighted 0.75 per half‐day shift. This quantitative framework strives to balance clinical duties fairly and transparently. For example, a physicist with a 60% clinical load, 40% research, teaching, and administrative commitment, and no planned time off would have a target of 12 FTE in a 20‐day scheduling block, possibly fulfilled by 9 general dosimetry shifts and 4 half‐day SBRT/SRS shifts. If another physicist with the same clinical percentage had five days of planned time off, their assigned workload would be proportionally reduced, for instance, to 9 FTE, possibly comprised of 6 community practice and 4 SBRT/SRS coverage slots. By centralizing this process and integrating all sources of effort, the team ensures non‐clinical activities, time off, and individual preferences are consistently factored into shift assignments.

### 2.5 Technical framework

While standardized FTE assignments provide a foundation for equitable workload distribution, the process of translating these targets into actual shift assignments presents considerable complexity. Our approach combines a streamlined, user‐friendly data input process with a robust optimization strategy that models the scheduling problem as a constrained binary integer optimization problem. Linear equality and inequality constraints are used to encode coverage requirements, FTE‐based shift targets, availability, and sequencing rules, while a nonlinear preference‐based penalty function is minimized using a genetic algorithm implemented in MATLAB.

#### 2.5.1 Input collection and preference encoding

Physicist availability and shift preferences are captured using a custom Excel graphical user interface (GUI), designed to resemble a color‐coded calendar spanning each two‐month scheduling block. Each half‐day clinical slot can be marked as:

- 0 (Red): Unavailable
- 0.5 (Yellow): Available, but not preferred
- 1 (Green): Available
- 2 (Bright Green): Available, and preferred

This interface supports the entry of complex patterns, allowing users to specify days off, dedicated research days, and individualized scheduling requests with a few clicks. Clinical days begin unmarked (gray) and are updated as selections are made, providing instant feedback through color coding. Once completed, these preference matrices, alongside the FTE‐derived target number of shifts for each individual, are exported for optimization. These inputs define both the potential assignments (availability) and the desirability of each shift placement (preference).

#### 2.5.2 Optimization design and algorithmic implementation

After collecting availability and preference data, all inputs are imported into MATLAB for scheduling optimization. Each possible assignment of a physicist to a half‐day or full‐day slot is represented as a binary decision variable in a constrained binary integer optimization framework:

$$\;x_{ijk}=\left\{\begin{array}{cc} 1, & \mathrm{if}\;\mathrm{physicist}\;i\;\mathrm{is}\;\mathrm{assigned}\;\mathrm{to}\;\mathrm{service}\;\\ & j\;\mathrm{during}\;\mathrm{time}\;\mathrm{slot}\;k\\ 0, & \mathrm{otherwise}. \end{array}\right.$$

The problem formulation is guided by several strict operational constraints, including adherence to individual FTE‐based shift targets, assignment of only one shift per person per day, and a heavy penalty for scheduling an early morning shift directly after a late afternoon/evening assignment for the same physicist. Assignments are further restricted by availability, so shifts cannot be given to anyone marked as “unavailable” on their input sheet. Computationally, an availability matrix is defined as

$$a_{ik}=\left\{\begin{array}{cc} 1, & {\mathrm{if}\;\mathrm{Preference}}_{ik}>0\\ 0, & {\mathrm{if}\;\mathrm{Preference}}_{ik}=0, \end{array}\right.$$

and assignments are constrained such that

$x_{ijk}\leq a_{ik}.$

Thus, shifts marked as unavailable are excluded as hard constraints. Additional linear constraints ensure that each required shift is assigned to exactly one physicist,

$$\;\sum_{i}x_{ijk}=1,$$

for each service and time slot, and that each physicist receives their target clinical workload,

$$\;\sum_{j,k}x_{ijk}=T_{i}\;,$$

where $T_{i}$ is the target number of assignments for each physicist, derived from the FTE‐weighted model. The model's objective function focuses on maximizing the alignment between scheduled shifts and individual preferences, while discouraging undesirable scheduling patterns. This is accomplished by minimizing a fitness function composed of a logarithmic preference penalty, a large sequencing penalty, and a smaller schedule‐spread term:

$$F\;\left(x\right)=F_{\mathrm{pref}}\;\left(x\right)+\lambda_{\mathrm{seq}}F_{\mathrm{seq}}\left(x\right)-\lambda_{\mathrm{spread}}F_{\mathrm{spread}}\left(x\right).$$

**FIGURE 1:**
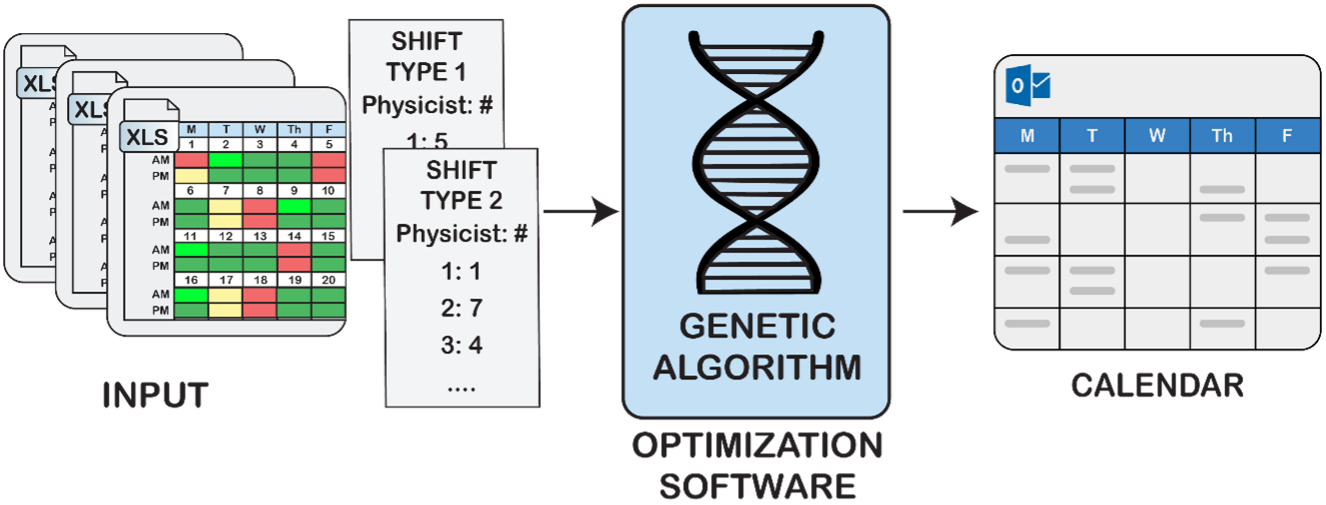
Flow diagram for the system, illustrating Excel spreadsheet inputs and target shift type assignments for each individual, the genetic algorithm‐based optimizer, and the final Outlook calendar output.

The penalty function is calculated as

$$F_{\mathrm{pref}}\left(x\right)=\sum_{i,j,k:x_{ijk}=1}\mathrm{lo}g_{10}\left(\frac{1}{p_{ik}}\right)$$

where $x_{ijk}$ denotes an assigned shift and $p_{ik}$ is the preference weight for each physicist and time slot. Lower‐preference available shifts therefore incur larger penalties than highly preferred shifts. The sequencing term $F_{\mathrm{seq}}$ penalizes assignment of an early morning shift immediately after a late afternoon/evening assignment for the same physicist, while $F_{\mathrm{spread}}$ rewards temporal distribution of assignments across the scheduling period. In the current implementation, $\lambda_{\mathrm{seq}}=1000$ and $\lambda_{\mathrm{spread}}=0.5$. This mechanism ensures that the final schedule prioritizes preferred shifts for each staff member, while still satisfying all operational constraints. Given the size and combinatorial complexity of the scheduling problem, as well as the nonlinear preference‐based fitness function, a conventional linear programming approach is not feasible. Therefore, we employ a genetic algorithm (GA), which iteratively generates and evolves candidate schedules using selection, crossover, and mutation. In each generation, candidate schedules are evaluated for feasibility and assigned a fitness score based on the fitness function. The GA proceeds until MATLAB's default stopping criteria are met, including a maximum stall generation limit of 50 generations and a function tolerance of 10^−6^, producing a schedule that balances clinical coverage requirements and staff preferences. A flowchart illustrating the major steps in the system is shown in Figure 1. Because runtime was not logged during initial clinical deployment, computational performance was assessed retrospectively by rerunning archived scheduling inputs from the six most recent scheduling periods using the finalized optimization workflow. Benchmarking was performed in MATLAB 2023b (version 23.2.0.2365128) on a 64‐bit Windows workstation running Microsoft Windows 10, with an Intel Core i5‐8500 CPU @ 3.00 GHz and 8 GB RAM. The GA used a population size of 200, elite count of 100, and no parallel computation. Termination occurred according to MATLAB's default GA stopping criteria. Runtime and generation counts were recorded for each rerun scheduling period.

#### 2.5.3 Scheduling workflow and output

After the optimal schedule is generated and reviewed, the assignments are returned to the Excel interface for final confirmation. To streamline integration with internal communication and patient care systems, the finalized schedule is then converted directly into Outlook calendar appointments using a custom Excel Visual Basic script. This export ensures that each staff member receives individual shift assignments in their personal Outlook calendar, while all scheduled clinical coverage is simultaneously reflected in the division's shared clinical calendar. By leveraging the native interoperability between Excel and Outlook, this workflow aims to eliminate manual data entry, reduce scheduling errors, and ensure that all participants have accurate access to coverage information.

### 2.6 Evaluation and analysis

Outcomes of the scheduling system implementation were assessed using both quantitative and qualitative methods. For each scheduling cycle, we retrospectively reviewed whether the algorithm was able to produce a feasible schedule that satisfied all hard constraints, including full clinical coverage, fulfillment of FTE targets, and adherence to individual staff availability. We tracked the distribution of shifts across the different preference categories to determine how well assignments aligned with user input and noted any cases where manual adjustments were required after the automated schedule was finalized. In addition to these operational metrics, informal observations and feedback from team members and scheduling leads were gathered to provide insights into overall satisfaction, perceived fairness, and the administrative impact of the new process. Fairness was assessed operationally rather than through a formal metric. Specifically, we evaluated whether shift targets were adjusted for non‐clinical effort and planned time off, whether all users submitted preferences through the same standardized interface, and whether final assignments demonstrated high preference concordance without assigning unavailable shifts. Due to the pragmatic nature of this clinical implementation, there was no formal collection of baseline or pre‐intervention data, and no controlled experimental design was employed. All assessments were based on retrospective schedule records and routine staff communications.

## 3 RESULTS

### 3.1 Implementation, usage, and preference concordance

The automated, preference‐based scheduling system was implemented in October 2023. The October–December 2023 period functioned as an implementation and refinement phase, with full adoption and routine data collection starting in January 2024. Quantitative results are reported from January 2024 through February 2026. Over this period, a total of 4980 clinical shifts were scheduled across fifteen blocks (Table 2). Each block spanned one to two months and included all physics faculty, staff, and residents participating in clinical coverage. The varying number of total shifts assigned per scheduling block reflects differences in clinical workdays, which were influenced by factors such as holidays and varying operational needs (e.g., no brachytherapy clinical coverage needed during HDR afterloader replacement and commissioning). Additionally, the brachytherapy service was incorporated into the optimization system beginning in March 2025. Prior to this, brachytherapy coverage was scheduled separately, and those shifts were excluded from the analyzed pool. Across all scheduling blocks, team members made a total of 25,796 shift selections. Of these, 17% were marked as preferred, 43% as available, 13% as non‐preferred, and 26% as unavailable. This distribution indicates that, while most selections reflected willingness to cover available shifts, a substantial proportion of selections included specific preferences or exclusions for certain shifts. Across the full study period, 95% of shifts were scheduled according to user preference and availability, with 2091 scheduled shifts (42%) marked as preferred and 2642 shifts (53%) as available. Only 5% of shifts (247 assignments) were designated as non‐preferred, while no team members were assigned to shifts they had indicated as unavailable during the entire evaluation period. Block‐by‐block results demonstrated consistently high rates of preferred or available scheduling (range: 86.8%–98.1%). The proportion of non‐preferred shift assignments peaked in early and mid‐2024, reaching a maximum of 13.2% in January–February 2025 and 12.3% in September–October 2024. The transient increases in non‐preferred assignments were likely multifactorial. September–October 2024 coincided with the highest proportion of shifts marked as non‐preferred by users (19%, compared with a median of 12% across scheduling periods), whereas January–February 2025 did not show a similar increase in non‐preferred selections. This suggests that non‐preferred assignment rates were influenced not only by preference‐entry patterns, but also by interacting constraints such as planned absences, service‐specific eligibility, uneven availability, and coverage requirements. In subsequent blocks, the rate of non‐preferred assignments declined steadily, stabilizing below 4% in the current implementation. This suggests both effective system optimization and increased alignment with staff input over time. Furthermore, manual corrections and post‐schedule swaps were rare and did not affect the integrity of the system's logic. In rare circumstances, certain scheduling rules required modification to ensure complete coverage. For example, there were occasional instances where an individual was assigned both a morning and afternoon half‐day shift on the same day despite the default restriction against this practice. Such exceptions were made only when feasible solutions respecting all constraints could not be identified, and assignments still aligned with the staff member's marked availability or preference. It is noteworthy that, under the prior non‐automated scheduling model, staff were routinely compelled to cover both AM and PM shifts, or similar undesirable combinations, in the same day, with little regard to individual preferences or workload balance. Retrospective benchmarking of the six most recent two‐month scheduling blocks demonstrated practical runtime for clinical use. Total runtime per block was a median of 35.3 min (range: 31.9–41.7 min), and individual genetic algorithm calls terminated after a median of 138 generations (range: 63–327 generations). Table 2 provides a full summary of shifts assigned, scheduling block duration, and assignment breakdowns by category.

**TABLE 2:** Shift assignment summary by scheduling block (Jan 2024–Feb 2026).

Scheduling block	Total shifts	Preferred	Available	Non‐preferred	Unavailable	Preferred or available
Jan–Feb 2026	400	148 (37.0%)	238 (59.5%)	14 (3.5%)	0	386 (96.5%)
Nov–Dec 2025	417	148 (35.5%)	257 (61.6%)	12 (2.9%)	0	405 (97.1%)
Sept–Oct 2025	431	171 (39.7%)	248 (57.5%)	12 (2.8%)	0	419 (97.2%)
July–Aug 2025	374	145 (38.8%)	220 (58.8%)	9 (2.4%)	0	365 (97.6%)
May–June 2025	430	217 (50.5%)	205 (47.7%)	8 (1.9%)	0	422 (98.1%)
Mar–Apr 2025	450	232 (51.6%)	202 (44.9%)	16 (3.6%)	0	434 (96.4%)
Jan–Feb 2025	356	97 (27.2%)	212 (59.6%)	47 (13.2%)	0	309 (86.8%)
Nov‐Dec 2024	378	164 (43.4%)	190 (50.3%)	24 (6.3%)	0	354 (93.7%)
Sept‐Oct 2024	389	161 (41.4%)	180 (46.3%)	48 (12.3%)	0	341 (87.7%)
July–Aug 2024	345	131 (38.0%)	190 (55.1%)	24 (7.0%)	0	321 (93.0%)
June 2024	160	77 (48.1%)	76 (47.5%)	7 (4.4%)	0	153 (95.6%)
Apr–May 2024	352	190 (54.0%)	153 (43.5%)	9 (2.6%)	0	343 (97.4%)
March 2024	178	78 (43.8%)	93 (52.2%)	7 (3.9%)	0	171 (96.1%)
Feb 2024	160	56 (35.0%)	98 (61.3%)	6 (3.8%)	0	154 (96.3%)
Jan 2024	160	76 (47.5%)	80 (50.0%)	4 (2.5%)	0	156 (97.5%)
**Total**	**4980**	**2091 (42.0%)**	**2642 (53.1%)**	**247 (5.0%)**	**0**	**4733 (95.0%)**

### 3.2 Operational outcomes and user feedback

**FIGURE 2:**
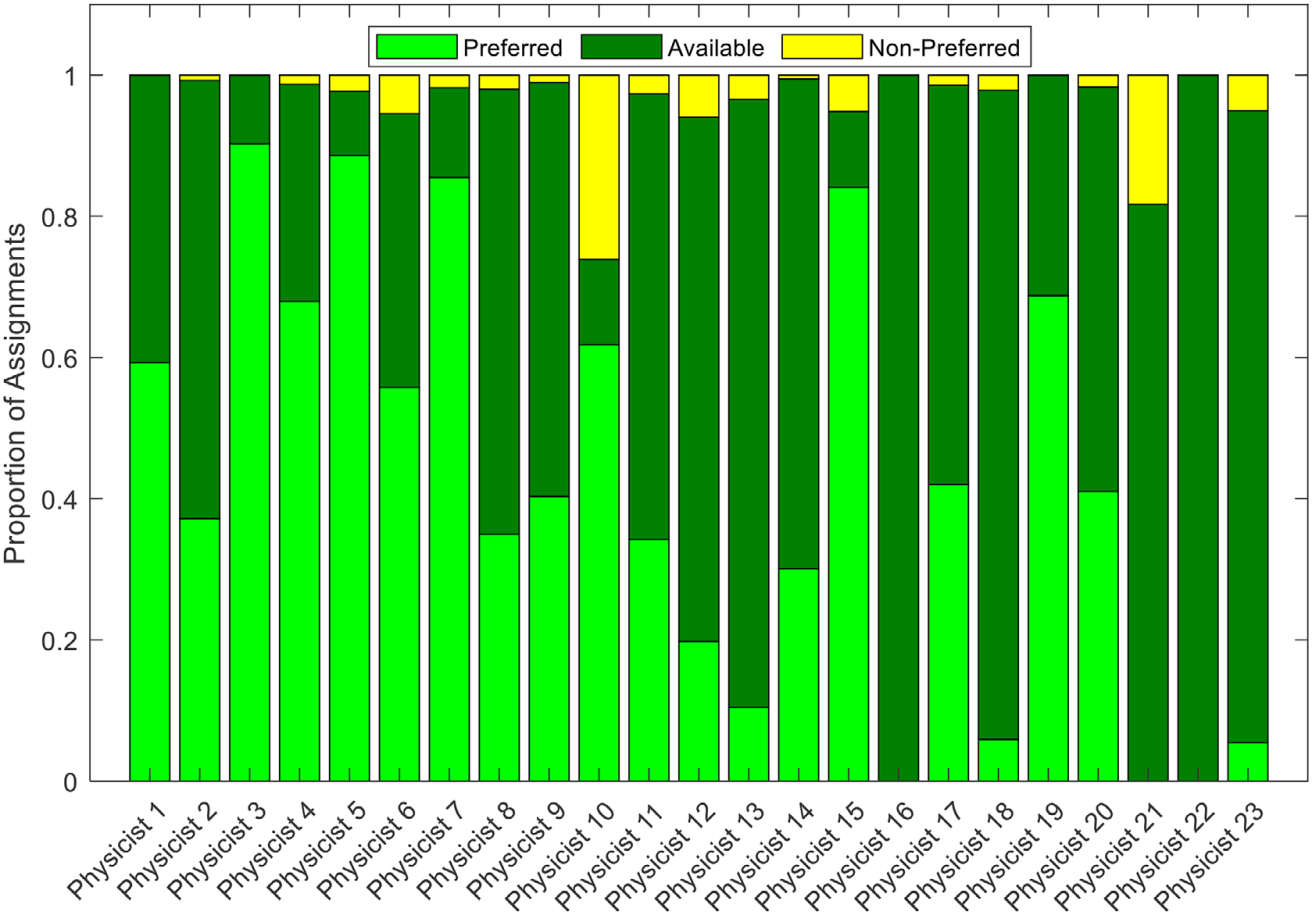
Bar plots showing preferred (bright green), available (green), and non‐preferred (yellow) shift assignments for each anonymized staff member over the analyzed period.

Although no formal measurement of scheduling efficiency or team satisfaction was conducted prior to implementation, several process metrics and anecdotal observations support the impact of the new scheduling framework. Figure 2 shows, for each physicist, the proportion of their shifts assigned as preferred, available, or non‐preferred. In general, physicists received shift assignments in a manner consistent with FTE‐adjusted targets and user‐entered preferences, with most team members consistently assigned to preferred or available shifts. Notably, Physicists 10 and 21 displayed higher‐than‐average proportions of non‐preferred shift assignments relative to other staff (Figure 2). However, both individuals routinely selected the non‐preferred option at a higher rate than their peers, resulting in a naturally elevated baseline. Anecdotal feedback from these users indicated satisfaction with the scheduling system and, especially, its flexibility. For example, Physicist 10 received a substantial fraction of assignments designated as preferred, suggesting that frequent use of the preference options allowed for effective tailoring of their schedule. Although baseline administrative time and schedule‐swap frequency were not prospectively recorded, scheduling leads reported that post‐publication swaps and manual interventions became infrequent after implementation, with most blocks requiring either zero or just a few minor post‐publication adjustments for late‐scheduled time off or unanticipated events. Feedback from physicists and scheduling leads indicates marked improvement in perceived fairness and transparency. Users report increased satisfaction with assignment predictability and a reduction in scheduling conflicts. Department leaders note a substantial reduction in administrative time spent resolving scheduling disputes, with completion of each cycle (from preference finalization to schedule dissemination) routinely achievable within a few days instead of several weeks.

## 4 DISCUSSION

The results of this study demonstrate that an automated, preference‐driven scheduling model, optimized using a genetic algorithm, is highly feasible for use in an academic medical physics environment. Our approach reliably produced individualized shift assignments that respected personal preferences and enforced strict operational constraints, yielding high rates of preferred or available scheduling and eliminating assignments to unavailable time slots.

Genetic algorithm‐based optimization has achieved notable success in workforce scheduling across various sectors. Previous work has addressed complex environments such as nurse rostering,^6^, ^9^ airline crew assignment,^7^ and multiprofessional healthcare teams.^8^, ^9^, ^10^, ^11^, ^12^, ^13^, ^14^, ^15^, ^16^, ^17^ These models typically leverage the flexibility of genetic algorithms to encode multiple constraints, optimize over large search spaces, and balance operational needs with individual preferences. Compared to approaches using mathematical programming or heuristic manual scheduling, genetic algorithms provide improved adaptability and scalability, especially when handling evolving constraints, new staff members and service types, and diverse individual preferences.

In this context, medical physics represents an especially compelling application for genetic algorithm scheduling. Clinical physicists face a distinct combination of multidisciplinary roles, variable shift types, diverse service areas, and frequent changes in specialty coverage. The need to balance clinical service, research obligations, education, and administrative duties further complicates the scheduling landscape. Our results show that the genetic algorithm‐based model is robust to these challenges: not only did the system accommodate individualized preferences and dynamic constraints, it also successfully integrated new services (such as brachytherapy) and adjusted for institutional changes (e.g., holidays or evolving clinical coverage requirements).

While commercial vendor solutions for clinical scheduling are available, our institution's contracted vendor was unable to smoothly incorporate individual preferences into the schedule generation process. Availability features, though technically supported, were cumbersome to configure on a day‐by‐day basis. In addition, setting up rules for shift sequencing (such as avoiding early morning following late afternoon) was not straightforward. In contrast, the flexibility of our automated framework allows staff to express nuanced preferences for specific shifts, such as marking some afternoons with a lower preference to enable activities like addressing childcare needs or attending regional professional group meetings, without unduly burdening colleagues. For example, using the *non‐preferred* setting lets a physicist indicate a shift is simply less desirable for them, and if another team member selects *available* or *preferred*, the system can allocate shifts concordantly with no additional cost or coverage gap. This type of day‐to‐day adaptability and personalization is often lacking in vendor‐driven systems, and our experience underscores its value in promoting work‐life integration and overall satisfaction without compromising coverage. Additionally, the software provides a flexible framework for on‐going development and to address specific needs. Features currently under investigation include “remote‐only” availability, which would allow physicists to specify when they can contribute to tasks that do not require onsite presence. In addition, when specific coverage is needed, such as a particular physicist assigned to certain tasks on certain days, these can be pre‐specified, allowing the system to optimize around those constraints. This flexibility helps maintain clinical coverage while supporting individual personal and professional needs. AI‐ or machine learning‐assisted scheduling tools may also be explored as complementary or alternative approaches, particularly for identifying preference patterns or anticipating scheduling bottlenecks; however, such methods would require transparent handling of hard clinical constraints, preservation of user‐entered preferences, and prospective validation before clinical use.

The specific service categories and FTE adjustments used in this implementation reflect our institutional structure, but the general framework may be adaptable to other practice models. Smaller departments or community‐based practices could simplify the number of service categories, reduce the number of constraints, or base targets primarily on clinical FTE and service expertise. This broader applicability was not evaluated in the present study; however, the core workflow of structured preference entry, workload target assignment, constrained optimization, and calendar export does not inherently depend on a specific academic staffing model.

Finally, while the implementation has been successful and sustained over two years, the project was initiated as a pragmatic clinical solution rather than a controlled study. As such, formal satisfaction surveys, perceived‐fairness ratings, burnout assessments, retention outcomes, and quantitative pre/post measures of workflow efficiency were not collected. Future work may include structured collection of these metrics, as well as expanded comparative analyses with alternative scheduling models.

## 5 CONCLUSION

We developed and deployed a preference‐integrated scheduling system that combines standardized FTE‐weighted service targets with genetic algorithm optimization for clinical shift scheduling. Over two years of routine use, the system consistently produced schedules that met clinical coverage requirements, honored stated availability, and minimized non‐preferred assignments, while improving fairness and reducing the administrative burden of schedule construction. This approach is practical and readily implementable for medical physics teams managing complex clinical staffing needs.

## AUTHOR CONTRIBUTIONS

**Benjamin S. Rosen**: Conceptualization; methodology; software development; implementation; statistical analysis; data visualization; writing—original draft; writing—review and editing. **Zheng Zhang**: Methodology; software development; technical validation; literature review; writing—review and editing. **Karolyn M. Hopfensperger**: Clinical implementation; user testing; literature review; workflow refinement; results interpretation; writing—review and editing. **Kelly C. Paradis**: Clinical implementation; user testing; workflow refinement; results interpretation; writing—review and editing. **Choonik Lee**: Methodology; software development; coding; technical validation; writing—review and editing. **Lana Critchfield**: Clinical implementation; user testing; workflow refinement; results interpretation; writing—review and editing. **Ikechi Ozoemelam**: Clinical implementation; user testing; workflow refinement; results interpretation; writing—review and editing. **Charles K. Matrosic**: Clinical implementation; user testing; workflow refinement; results interpretation; writing—review and editing. **Christopher Kehayias**: Clinical implementation; user testing; workflow refinement; results interpretation; writing—review and editing. **Scott W. Hadley**: Clinical implementation; user testing; workflow refinement; results interpretation; writing—review and editing. **James M. Balter**: Clinical implementation; user testing; workflow refinement; results interpretation; writing—review and editing. **Claire Zhang**: Clinical implementation; user testing; workflow refinement; results interpretation; writing—review and editing. **Lise Wei**: Clinical implementation; user testing; workflow refinement; results interpretation; writing—review and editing. **Seyyedeh Azar Oliaei Motlagh**: Clinical implementation; user testing; workflow refinement; results interpretation; writing—review and editing. **Alexandar Moncion**: Clinical implementation; user testing; workflow refinement; results interpretation; writing—review and editing. **Dale W. Litzenberg**: Clinical implementation; user testing; workflow refinement; results interpretation; writing—review and editing. **Cory Knill**: Clinical implementation; user testing; workflow refinement; results interpretation; writing—review and editing. **Maria G. Ditman**: Clinical implementation; user testing; workflow refinement; results interpretation; writing—review and editing. **Elizabeth L. Covington**: Clinical implementation; user testing; workflow refinement; results interpretation; writing—review and editing. **Charles S. Mayo**: Clinical implementation; user testing; workflow refinement; results interpretation; writing—review and editing. **Sean P. Devan**: Clinical implementation; user testing; workflow refinement; results interpretation; writing—review and editing. **Martha M. Matuszak**: Conceptualization; methodology; clinical implementation; supervision; results interpretation; writing—review and editing. **Joann I. Prisciandaro**: Conceptualization; methodology; clinical implementation; supervision; results interpretation; writing—review and editing.

## CONFLICT OF INTEREST STATEMENT

The authors declare no conflicts of interest.

## ETHICS STATEMENT

This project was conducted as an operational workflow improvement and retrospective evaluation of clinical scheduling processes. No patient data were analyzed, and no intervention involving patients or research participants was performed. Formal ethical approval was not required.

## GENERATIVE AI STATEMENT

During the preparation of this work, the authors used UM‐GPT‐5.5, the University of Michigan's secure, HIPAA‐compliant large language model, to assist with language editing and improve clarity. After using this tool, the authors reviewed and edited the content as needed and take full responsibility for the content of the published article.

## Data Availability

The data and software supporting the findings of this study are available from the corresponding author upon request.
